# Circulating tumor cells as a new predictive and prognostic factor in patients with small cell lung cancer

**DOI:** 10.7150/jca.35308

**Published:** 2020-02-03

**Authors:** Pei-Pei Wang, Si-Hong Liu, Cun-Te Chen, Lin Lv, Dan Li, Qiong-Yao Liu, Guo-Long Liu, Yong Wu

**Affiliations:** 1 Department of Oncology, Guangzhou First People's Hospital, School of Medicine, South China University of Technology; 2 Department of Orthopaedics, Guangzhou First People's Hospital, School of Medicine, South China University of Technology; 3 Department of Hematology, Guangzhou First People's Hospital, School of Medicine, South China University of Technology; 4 Guangzhou First People's Hospital, Guangzhou Medical University, Guangzhou, Guangdong, People's Republic of China, 510180

**Keywords:** circulating tumor cell, prognosis, nomogram, risk stratification, SCLC

## Abstract

**Background**: Small cell lung cancer (SCLC) is the most malignant type of lung cancer characterized by rapid progression, early metastasis and recurrence. In recent years, circulating tumor cells (CTCs) were found to play an important role in tumor invasion, metastasis, recurrence and prognosis.

**Methods**: CTCs were detected in 138 patients with newly diagnosed SCLC from January 2012 to December 2018. Nomogram prediction models were constructed based on prognostic factors screened by multivariate Cox regression analysis and the risk stratification of SCLC patients were performed on basis of nomogram points. A total of 108 patients from January 2012 to December 2016 were assigned to a training group, and 30 patients from January 2017 to December 2018 were included into the validation group for nomogram analysis. This study was approved by ethics committee of Guangzhou First People's Hospital and all subjects provided informed consent.

**Results**: The number of CTCs was associated with age, lymph node metastasis (N), distant metastasis (M), TNM staging, and NSE. The high number of CTC predicted adverse prognosis, and the AUC of time-dependent ROC curve was all high than 0.5. In the training group, after multivariate COX regression screening, the factors in the median survival time (MST) and overall survival (OS) nomogram prediction models were age, TNM, CTC, NSE and treatment mode. The C-index of the nomograms in internal validation for MST and OS was 0.813 and in external validation for MST and OS were 0.885. The AUC of ROC curves for nomogram were high than 0.5. Finally, risk stratification could be effectively performed on the basis of nomogram points.

**Conclusions**: CTC can be served as a predictive and prognostic factor for SCLC, and the nomogram models constructed by CTC and multiple clinical parameters can comprehensively predict the prognosis of SCLC patients and perform risk stratification.

## Introduction

Small cell lung cancer (SCLC) is a type of highly malignant tumor with neuroendocrine function, which can cause distant metastasis in the early stage of the disease. Although SCLC is sensitive to radiation and chemotherapy, many treatments fail due to recurrence and metastasis 1. Patients with SCLC have a very poor prognosis, the median survival time is 8 ~ 12 months, and the 5-year survival rate is about 1% ~ 5% 2. Studies have shown that SCLC patients with high CTC levels have adverse outcome. At the same time, SCLC patients with high CTC levels after treatment or relapse have a worse survival than those with low CTC levels 3. In SCLC, CTCs can be used as a predictive and prognostic factor 4. By detecting circulating tumor cells, tumor markers and imaging examinations, SCLC patients can be diagnosed, tested for therapeutic effects and evaluated for prognosis. A variety of CTC assays are currently used in clinics, and the Cell Search system has been approved by the FDA to predict progression-free survival (PFS) and overall survival (OS) of various cancers 5-7.

The nomogram is a graph calculator based on multi-factor regression analysis. Nomogram can visually display the results of the regression model, which converts the contribution of each influencing factor to the outcome variable into a series of straight lengths or different values of the variables, and obtains predictive values of individual outcome time for visual prognostic prediction 8. A variety of clinicopathological parameters are usually used to monitor disease progression, predict survival prognosis, adjust individualized treatment options for patients. The nomogram models have been verified in predicting the prognosis of various cancers, such as cervical cancer, prostate cancer, and gastric cancer, and they can predict the survival time and survival probability of patients by integrating multiple influencing factors 9-11.

The prognosis of patients is roughly estimated by clinical indicators such as TNM staging, CTC testing and tumor marker detection. Compared with traditional single clinical indicators to evaluate the prognosis, this study can predict more accurately by integrating the nomogram model established by multiple clinical indicators, and it can effectively conduct risk stratification through the nomogram points of the patients, providing an important basis for personalized medicine.

## Materials and Methods

### Patient's clinicopathological features and nomogram model grouping

The clinical data of 138 patients with newly diagnosed small cell lung cancer from January 2012 to December 2018 in Guangzhou First People's Hospital were collected, including 124 males and 14 females, aged 45 ~ 88 years, with an average age of 65 years old. The clinical staging was based on the 8th edition of the International Association for the Study of Lung Cancer (IASLC) 12-14. The inclusion criteria were as follows: (1) Small cell lung cancer was newly diagnosed by pathological biopsy; (2) Complete sociodemographic data and laboratory test results; (3) Complete TNM staging by pathology, radiology, surgery or imaging; (4) Complete treatment data; (5) Complete follow-up information. The exclusion criteria were as follows: (1) Non-small cell lung cancer; (2) Unknown histological subtype of lung cancer. There were 5 cases in stage I, 9 cases in stage II, 36 cases in stage III, and 88 cases in stage IV. After obtaining the patient's informed consent, peripheral blood was taken to detect circulating tumor cells (CTCs) before treatment. In the treatment, cisplatin or carboplatin in combination with etoposide or irinotecan (4 ~ 6 cycles) and radiation were performed for patients who were unable to undergo complete surgical resection with limited stage SCLC, while cisplatin or carboplatin combination with etoposide or irinotecan (4 ~ 6 cycles) were conducted in extensive stage SCLC. All patients were followed up until March 1^st^, 2019. The median follow-up time for the surviving patients was 240 days (range, 4 ~ 1580 days). This study was approved by ethics committee of Guangzhou First People's Hospital and all subjects provided informed consent.

A total of 108 patients from January 2012 to December 2016 were assigned to a training group, and 30 patients from January 2017 to December 2018 were included into the validation group for nomogram analysis.

### Detection of circulating tumor cells

**Figure 1:**
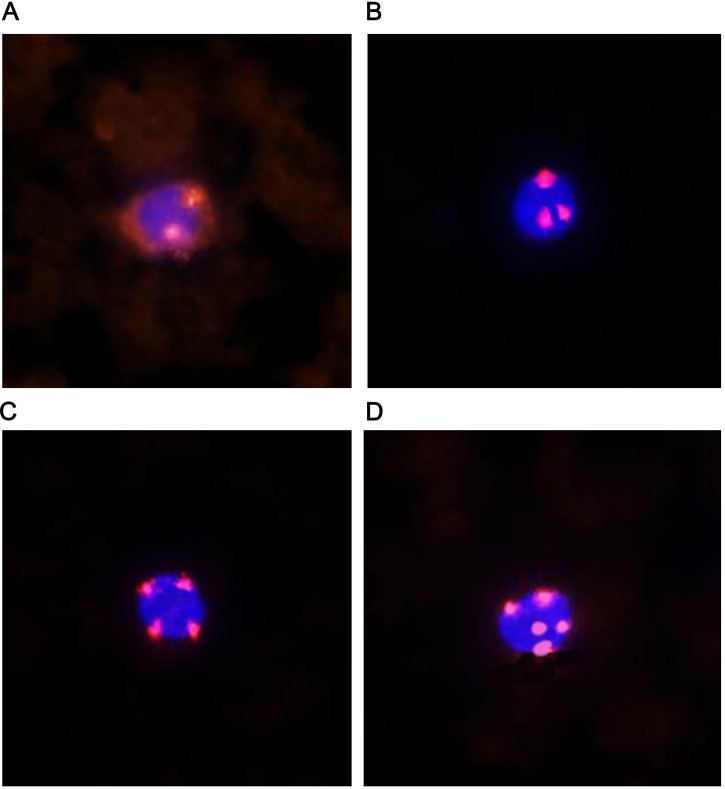
** Detection of normal cells and circulating tumor cells.** (A) DAPI positive, CEP8 negative (signal = 2) and CD45 positive normal cells. (B, C, D) DAPI positive, CEP8 positive (signal ≥ 3) and CD45 negative circulating tumor cells. DAPI, 4',6-diamidino-2-phenylindole; CEP8, chromosome 8 centromere probe; CD45, cell differentiation antigen 45.

CTC was detected by fluorescence *in situ* hybridization with chromosome 8 centromere probe (CEP8) in combination with CD45 immunofluorescence antibody technology (CD45-FISH) 15, 16. To prevent contamination of the epithelial cells during the venipuncture, the first 2 mL of blood was not collected. After that, 4 ~ 5 mL of venous blood was collected to ACD anticoagulant tube which contain anticoagulation. The plasma was removed within 24 hours, and the red blood cell lysis buffer was used to remove red blood cells. Isolation and enrichment of tumor cells was done by EpCAM-independent method magnetic beads. Cellular immunofluorescence was performed using a CD45 fluorescent antibody, and chromosome 8 centromere probe was used for fluorescence in situ hybridization (FISH). 4',6-diamidino-2-phenylindole (DAPI) labeled nuclei, CD45 labeled leukocytes, CEP8 labeled chromosome 8 centromeres. As judged by fluorescence microscopy, DAPI positive, CEP8 negative (signal = 2) and CD45 positive cells were normal cells. DAPI positive, CEP8 positive (signal ≥ 3) and CD45 negative cells were judged as circulating tumor cells (Fig. 1).

### X-tile for the optimal cut-points

X-tile (version 3.6.1, Yale University, New Haven, CT, USA) 17 is a new bioinformatics tool for determining the optimal cut-points for survival analysis of quantitative variables. The X-tile software tested all possible cut-points of the target data by a log-rank test of the highest χ^2^ value and lowest *p*-value. The X-tile plots were constructed by dividing the quantitative variables into three groups: Low, middle and high, which based on the optimal cut-points.

### Nomogram models construction

Univariate and multivariate Cox proportional hazard regression models were performed by using SPSS software (version 22.0, IBM, Chicago, IL) to screen the independent prognostic factors of clinical data for Median survival time (MST) and overall survival (OS) analysis. Clinically significant variables were further incorporated into the construction of MST and OS nomogram models. The foreign, rms, hmisc, lattice, survival, formula and ggplot2 packages in R (version 3.5.1, http://www.r-project.org/) were applied for nomogram models analysis. Model performance was evaluated by internal and external validation, which was showed by concordance index (C-index) and calibration curves using 1000 sample bootstrap. To further validated the predictive ability for nomogram model in training group, the AUC of time-dependent ROC curve was used for evaluation in R (version 3.5.1, http://www.r-project.org/). Finally, each patient was given a total point using standard points obtained from the nomogram models, which could predict MST and survival probabilities of SCLC patients. The median survival time (MST) was the time corresponding to a cumulative survival rate of 50%, indicating that only 50% of individuals can survive beyond this cut-point. Overall survival (OS) referred to the time from the start of the diagnosis of the disease to the death for any reason.

### Statistical analysis

Qualitative variables were categorized prior to modeling based on clinical experience and significance. For continuous variables, the optimal cutoff of CTC and age was obtained using X-tile software (version 3.6.1, Yale University, New Haven, CT, USA) 17. The time-dependent ROC curves were used for predictive ability of CTC in R (version 3.5.1, http://www.r-project.org/). Student's t-test, ANOVA and Chi-Square test (χ^2^ test) were performed to compare the significance of differences between groups, as appropriate. A two-tailed *p* value < 0.05 was considered statistical significance.

## Results

### Clinical characteristics

The clinical characteristics of the patients in the training and validation groups for MST and OS analysis were listed in Table 1. The result showed that there was no significant difference (*p*>0.05) for all clinical variables between the training group and the validation group, so all the variables could be used for following analysis.

### X-tile for the optimal cutoff of CTC and age

**Figure 2:**
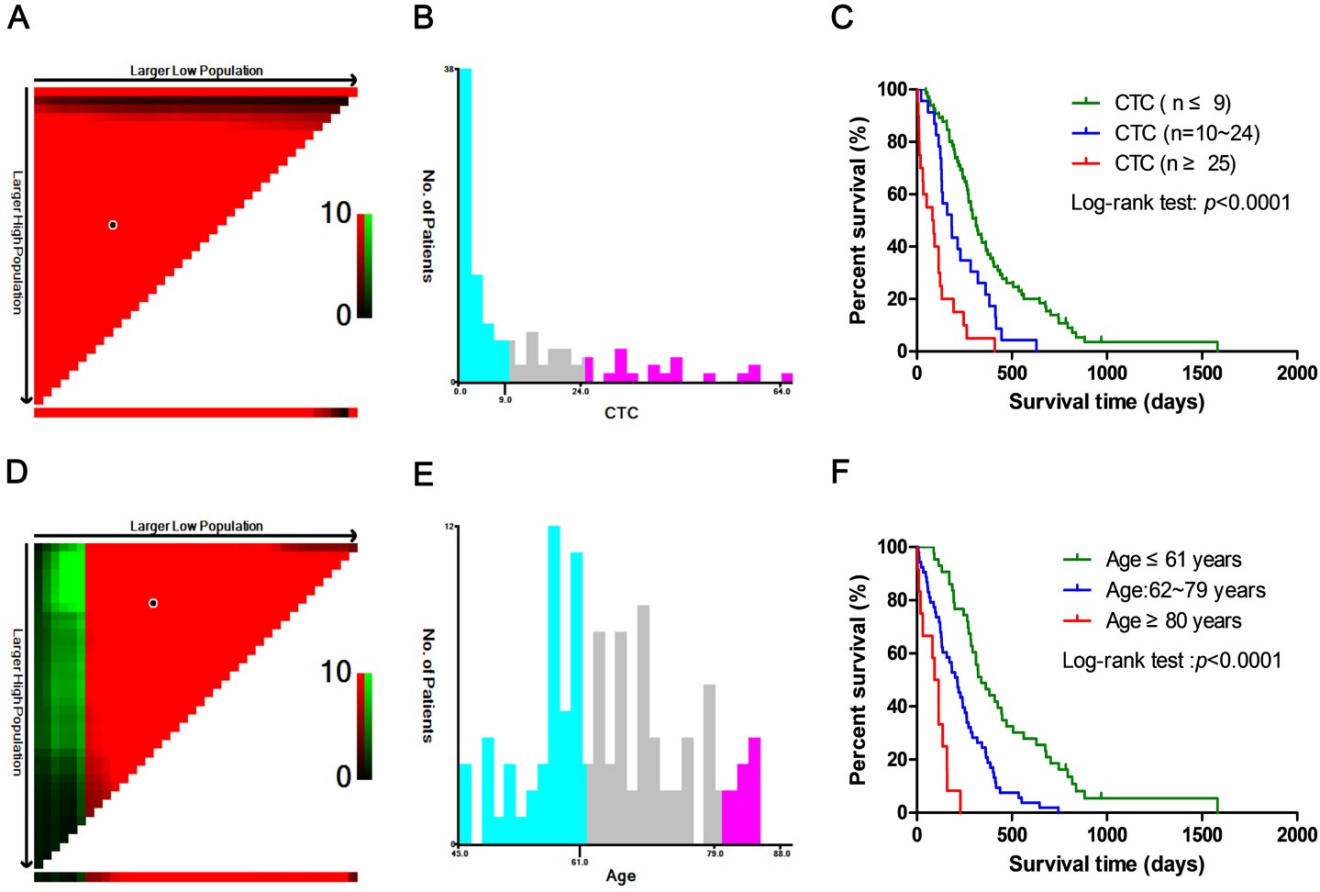
** The optimal cut-points of CTC and age obtained from X-tile software.** (A, D) The figure showed the optimal cut-points were generated from the log-rank χ^2^ values. The green color was positively correlated with survival rate, while the red color was the opposite. The brightest pixel (red or green) was the optimal cut-point. (B, E) Distribution of the numbers of patients was showed in different CTC and age groups based on the two optimal cut-points. (C, F) The survival curve was plotted on the basis of the optimal cut-points, which revealed significant differences.

X-tile software was used to determine the optimal cut-points of age and CTC in training group (n = 108), which was applied for univariate and multivariate Cox proportional hazard regression analysis, as well as nomogram models construction. As shown in Fig. 2, the optimal cut-points of CTC and age for analysis were ≤9, 10 ~ 24, ≥25 and ≤61, 62 ~ 79, ≥80 years old respectively, which indicated significant difference among cut-points.

### The relationship between CTC and clinical factors

**Figure 3:**
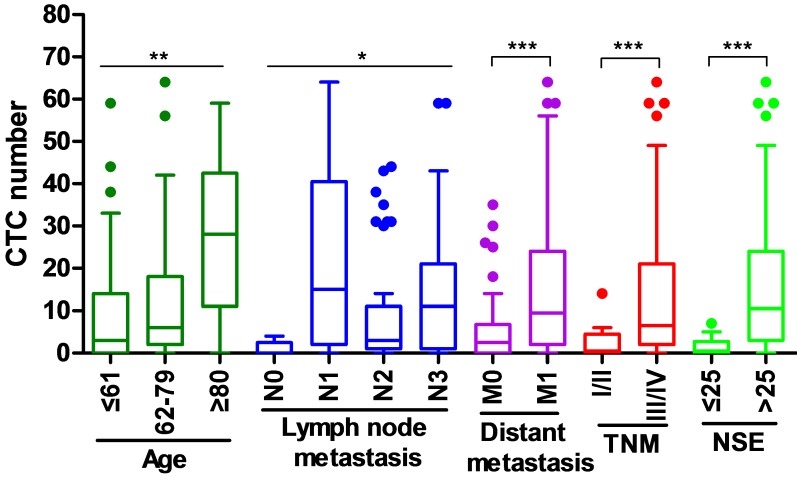
** The relationship between CTC and clinical factors in training group.** ^*^p<0.05, ^**^p<0.01, ^***^p<0.001.

The correlation between the number of CTC and the clinical parameters of newly diagnosed SCLC patients was investigated (Fig. 3). The higher numbers of CTC were observed in patients with old age (≥62 years), lymph node metastasis (N1-3), distant metastasis (M1), III/IV stage of TNM, and high expression of NSE (>25 ng/ml), while the numbers of CTC were lower in patients with young age (≤61 years), without lymph node metastasis (N0), without distant metastasis (M0), I/II stage of TNM and low expression of NSE (≤25 ng/ml).

### Cox regression analysis of training group

Univariate COX proportional hazard regression analysis for MST and OS suggested that there were significant differences in survival rates of age, T, N, M, TNM, CTC, NSE and treatment mode, which could be further included in multivariate COX regression analysis (Table 2). As shown in Table 2, multivariate COX proportional hazard regression models demonstrated that age, TNM, CTC, NSE and treatment mode were independent prognostic factors of SCLC in the OS analysis.

### Nomograms of SCLC for MST and OS

**Figure 4:**
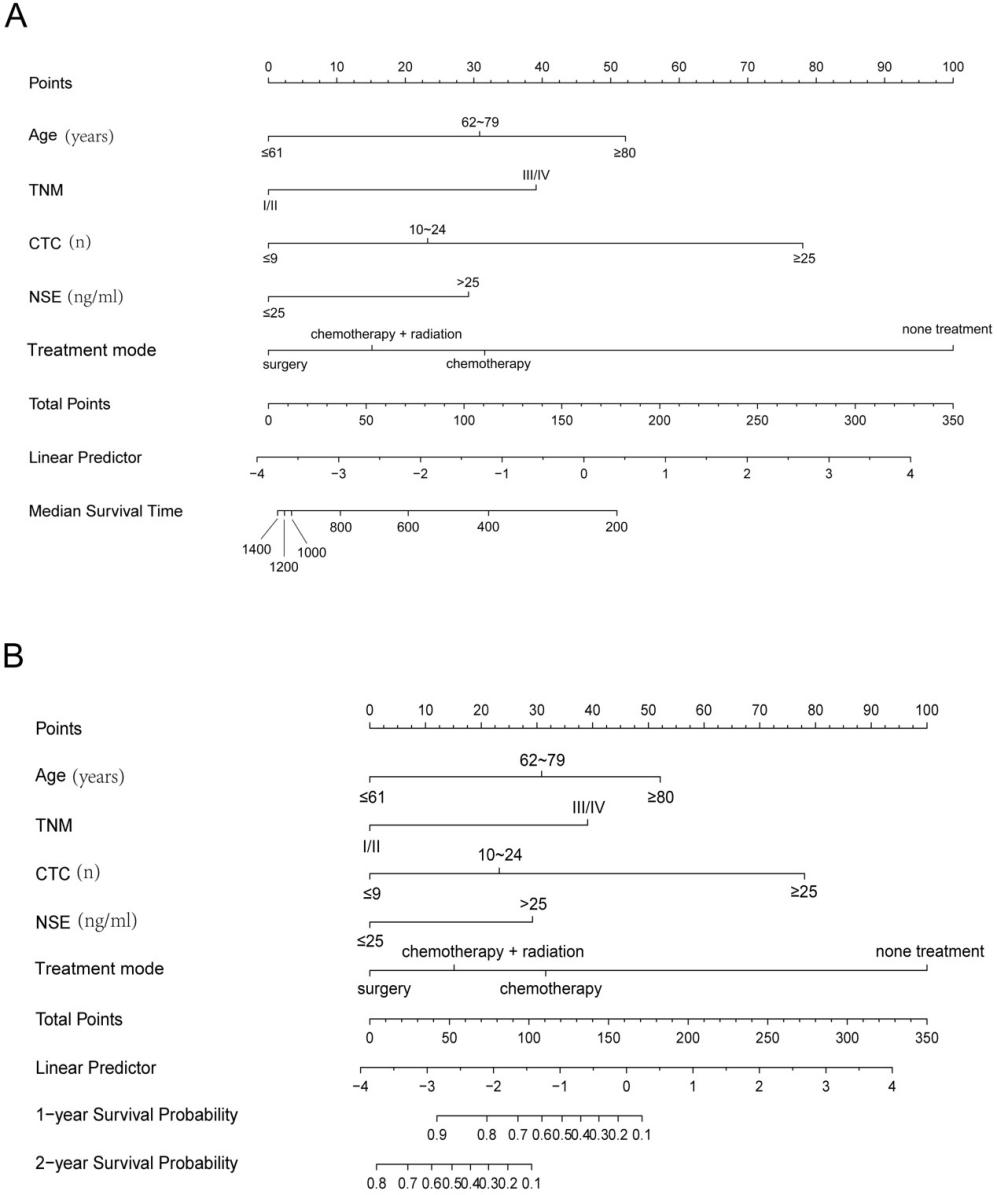
** Nomogram models for MST (A) and OS (B) of SCLC.** First of all, the covariate of each patient was given a point based on the nomogram. Then, the total points were obtained by gathering the given points of all covariates of a patient. Finally, the median survival time and the survival probabilities of 1- and 2-year OS corresponding to the total points could be showed by the nomogram. Additionally, a higher total point usually suggested a lower median survival time and a higher possibility of a lower predicted survival probability (MST and OS). MST, median survival time; OS, overall survival.

Clinical parameters after multivariate COX regression selection were channeled into the construction of training group nomograms (Fig. 4). Details of the labels for tick marks and points in nomograms were shown in Table 3.

### Internal and external validation

**Figure 5:**
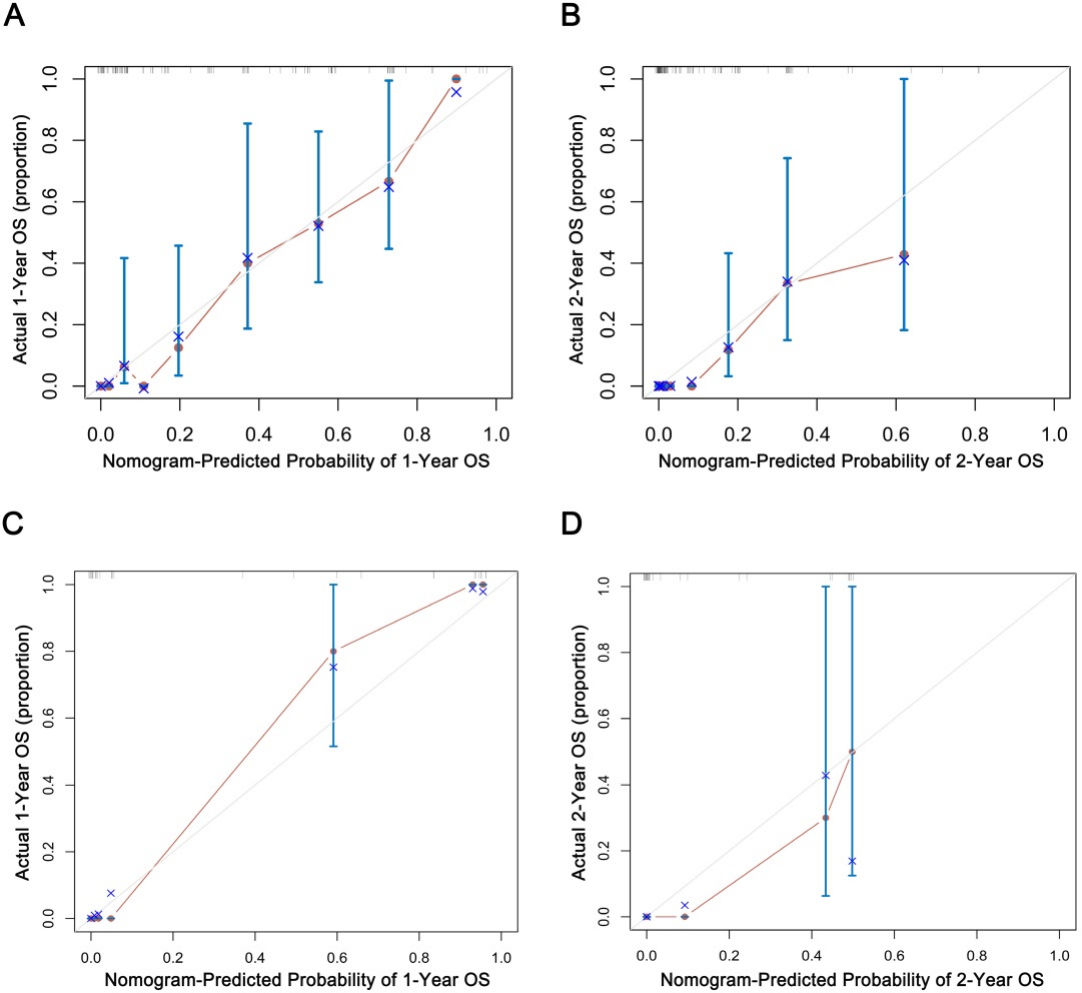
** Internal (A, B) and external (C, D) validation of nomograms in the training groups.** The predicted probabilities of 1- and 2-year were consistent with the actual survival proportions of patients with SCLC. OS, overall survival.

The C-index of 1000 sample bootstrap was 0.813 for the MST and OS predictive nomograms, which indicated nomograms for MST and OS shown relatively precise ability of discrimination. Further calibration curves manifested that the probability of predicted 1- and 2-year OS in nomograms were well consistency between the predicted outcome and actual observation (Fig. 5A, B).

In the external validation group, the C-index of predictive accuracy for MST and OS were 0.885 (Fig. 5C, D). The external calibration curves also illustrated good validation between predicted and observed 1- and 2-year OS. The discrimination and calibration validation of external group definitely certificated that nomogram models in this study could be comparatively accurate enough to predict the MST and OS probability of patients with SCLC.

### ROC curves for nomogram and CTC

**Figure 6:**
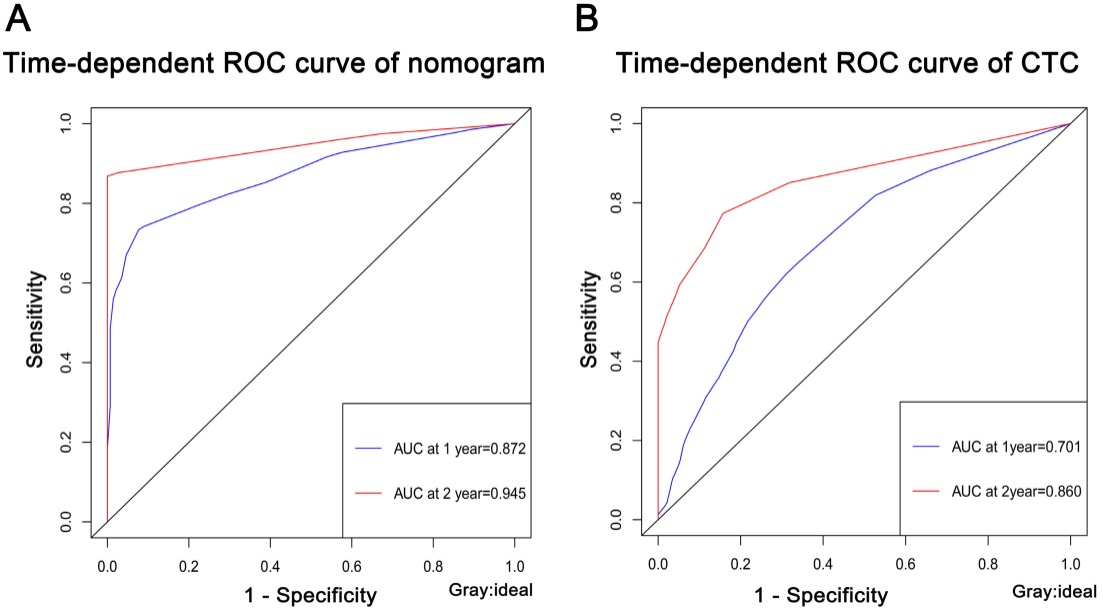
** The predictive ability for nomogram (A) and CTC (B) in training group.** The AUC of ROC curves were all high than 0.5. ROC, receive operating characteristic curve; AUC, area under the curve.

To further validated the predictive ability for nomogram and CTC in training group, the AUC of time-dependent ROC curve was used for evaluation. The 1- and 2-year of AUC for nomogram were 0.872 and 0.945, respectively (Fig. 6A). Furthermore, to clarify the predictive ability of CTC on the prognosis of SCLC, the AUC at 1- and 2-year were 0.701 and 0.860, respectively (Fig. 6B). The AUC of ROC curves indicated that the nomogram model had a more accurate prediction of the prognosis of patients with SCLC than CTC.

### Risk stratification in SCLC patients

**Figure 7:**
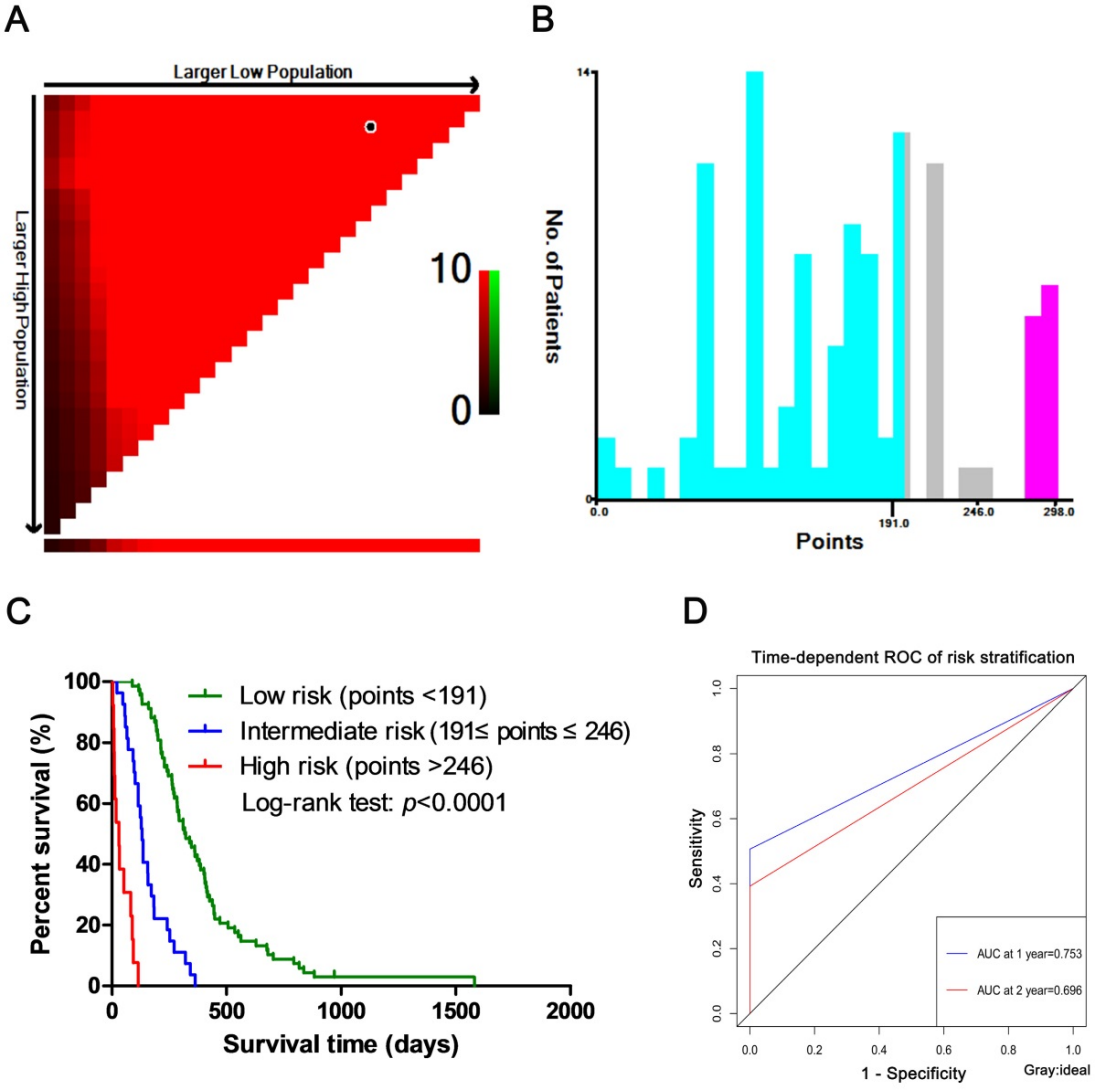
** Risk stratification in SCLC.** (A) The figure showed the optimal cut-points were generated from the log-rank χ^2^ values. The green color was positively correlated with survival rate, while the red color was the opposite. The brightest pixel (red or green) was the optimal cut-point. (B) The distribution of the numbers of patients was showed in different risk groups based on the two optimal cut-points. (C) The survival curve of risk stratification was plotted on the basis of the optimal cut-points, showing significant differences. (D) The predictive ability for risk stratification in training group. ROC, receive operating characteristic curve; AUC, area under the curve.

Nomogram models was constructed based on the weight of each clinical factor in the multivariate COX regression analysis. Then, the patients were divided into three groups (low-, intermediate- and high-risk groups) by using the X-tile tool to determine the optimal cut-points based on the nomogram points (Fig. 7A, B). The optimal cut-points of the nomogram points were <191, 191 ~ 246 and >246. As showed in Fig. 7C, the Kaplan-Meier curve for OS among the low-, intermediate- and high-risk groups showed significantly different (*p*<0.0001). At the same time, the AUC at 1- and 2-year were 0.753 and 0.696, respectively (Fig. 7D).

## Discussion

Small cell lung cancer accounts for 15% of new lung cancers each year, but due to its aggressive characteristics, it develops metastatic disease in the early stages of the disease, resulting in the highest mortality rate among all types of lung cancer 18. Circulating tumor cells enter the circulatory system after induction of epithelial-mesenchymal transition from the primary or metastatic lesion. Our studies showed that CTC counts were closely related to TNM stage, lymph node metastasis and distant metastasis, which indicated that CTC counts had a closely relationship with metastasis in SCLC patients. At the same time, we also found that CTC counts had a lot to do with other clinical factors such as age and serum tumor marker NSE. In this study, the higher the CTC count, the worse the patient's prognosis, and the CTC had a good diagnostic effect on the prognosis of SCLC patients, indicating that CTC could be used for prognosis prediction.

Many studies have found that the prognosis of small cell lung cancer can be predicted by detecting peripheral blood circulation tumor cells (CTCs) 3-4, 19. A study by Naito et al 3 has shown that SCLC patients with CTCs ≥8 have worse survival rate than those with CTCs <8. In our study, CTCs were divided into ≤9, 10 ~ 24 and ≥25, and SCLC patients with 10 ~ 24 and ≥25 CTCs had worse prognosis than those with ≤9 CTCs. Although studies by Hiltermann et al 4 and Normanno et al 19 also clarify that CTC is a factor affecting the prognosis of SCLC, none of them use CTC for individualized prediction of the prognosis in SCLC patients. In this study, CTC was used as one of the factors to predict individualized prognosis in SCLC patients, which had clinical utility.

As we known that in the course of clinical diagnosis and treatment, in order to monitor the progress of the disease, it is necessary to make a diagnosis and treatment response in real time, and better methods are needed to assess the disease impact. At the same time, the clinical indicators of the disease are not independent, and they are also affected by other factors. Many studies have used prognostic predictions for progression-free survival and overall survival of limited or extensive SCLC by TNM staging, serum tumor markers, or circulating tumor cells and their DNA derivatives to adjust treatment options or assess disease burden 20-23. Compared with other clinical prediction models, the nomogram model is more individualized, visual, and accurate in predicting the survival probability of each patient, which plays an increasingly important role in clinical evaluation of prognosis 24-26. In this study, we developed two prognostic nomogram models based on CTC, age, TNM staging, NSE and treatment mode to predict MST and OS in SCLC patients. Although the prediction probabilities of the nomogram models in the internal and external validation were not completely consistent with observation probabilities, the C-indexed were all higher than 0.8, which gave great prediction accuracy and repeatability. Further time-dependent ROC curve analysis of the nomogram models, AUC and C-indexes had high consistency, indicating that the models could provide more accurate and reliable predictive evaluation for SCLC patients.

We further performed risk stratification of SCLC patients on the basis of points derived from the nomogram mode, and the risk stratification for SCLC patients was well validated with the time-dependent ROC curve. According to the SCLC patients in the low-, intermediate- and high-risk groups, we could carry out targeted and individualized treatments to improve the overall survival rates of patients.

It is worth noting that the main limitation of this study is that the construction and validation of the nomogram models are based on a small number of patients in a single-center. More clinical data is still needed to validate the nomogram models of SCLC patients in order to perform risk stratification more reliably and accurately. Importantly, randomized controlled multi-center clinical trials are needed to obtain more favorable studies for the prognosis evaluation of circulating tumor cells in small cell lung cancer.

In summary, this study showed that CTC could be served as a predictive and prognostic factor for SCLC, and the nomogram models constructed by CTC and multiple clinical parameters could better predict the median survival time and survival probability of SCLC patients and perform risk stratification, which had clinical utility and relative reliability in SCLC patients.

## Figures and Tables

**Table 1:** Clinical characteristics of 138 SCLC patients.

Variables	Total	Training group	Validated group	*p*-value
**Number**	138	108	30	
**Age, mean±SD, years**	64.8±10.2	65.3±10.1	63.0±10.7	0.280
**Male, n (%)**	124 (89.9)	97 (89.8)	27 (90)	1.000
**Tumor invasion depth, n (%)**				0.172
T1	8 (5.8)	6 (5.5)	2 (6.7)	
T2	22 (15.9)	14 (13.0)	8 (26.7)	
T3	35 (25.4)	31 (28.7)	4 (13.3)	
T4	73 (52.9)	57 (52.8)	16 (53.3)	
**Lymph node metastasis, n (%)**				0.676
N0	8 (5.8)	5 (4.6)	3 (10.0)	
N1	27 (19.5)	21 (19.4)	6 (20.0)	
N2	59 (42.8)	48 (44.5)	11 (36.7)	
N3	44 (31.9)	34 (31.5)	10 (33.3)	
**Distant metastasis, n (%)**	87 (63.0)	68 (63.0)	19 (63.3)	0.970
**TNM stage, III/IV, n (%)**	124 (89.9)	98 (90.7)	26 (86.7)	0.755
**CTC, mean±SD, n**	13.1±15.9	12.5±15.5	15.3±17.4	0.387
**NSE, >25ng/ml, n (%)**	100 (72.5)	84 (77.8)	20 (66.7)	0.212
**Cyfra21-1, >3.3ng/ml, n (%)**	94 (68.1)	70 (64.8)	24 (80.0)	0.114
**CEA, ≥5ug/L, n (%)**	63 (45.7)	51 (47.2)	12 (40.0)	0.482
**SCC, ≥2.5ng/ml, n (%)**	10 (7.2)	7 (6.5)	3 (10.0)	0.795
**Treatment mode, n (%)**				0.169
chemotherapy	61 (44.2)	52 (48.1)	9 (30.0)	
chemotherapy + radiation	4 (2.9)	2 (1.9)	2 (6.7)	
surgery	5 (3.6)	3 (2.8)	2 (6.7)	
none treatment	68 (49.3)	51 (47.2)	17 (56.6)	

Abbreviations: SD, standard deviation; NSE, neuron-specific enolase; Cyfra21-1, cytokeratin 19 fragment; CEA, Carcinoembryonic antigen; SCC, squamous cell carcinoma associated antigen; CTC, circulating tumor cells.

**Table 2:** Univariate and multivariate cox regression analysis of 108 SCLC patients.

Variables	Univariate cox regression				Multivariate cox regression		
	HR	95%CI	p-value		HR	95%CI	p-value
**Age (years)**							
≤61	1.000	Referent			1.000	Referent	
62~79	2.470	1.580~3.861	<0.001***		2.215	1.336~3.672	0.002**
≥80	9.114	4.378~18.974	<0.001***		3.464	1.556~7.714	0.002**
**Gender**							
female	1.000	Referent					
male	1.111	0.593~2.083	0.743				
**Tumor invasion depth**							
T1	1.000	Referent					
T2	1.753	0.623~4.931	0.288				
T3	3.038	1.167~7.909	0.023*				
T4	3.447	1.362~8.724	0.009**				
**Lymph node metastasis**							
N0	1.000	Referent					
N1	2.979	1.013~8.760	0.047*				
N2	4.477	1.579~12.694	0.005**				
N3	4.185	1.469~11.923	0.007**				
**Distant metastasis**							
M0	1.000	Referent					
M1	2.248	1.481~3.413	<0.001***				
**TNM stage**							
I/II	1.000	Referent			1.000	Referent	
III/IV	4.788	1.863~12.308	0.001**		3.179	1.406~7.184	0.005**
**CTC(n)**							
≤9	1.000	Referent			1.000	Referent	
10~24	1.881	1.151~3.074	0.012*		1.445	0.837~2.497	0.187
≥25	5.375	3.124~9.248	<0.001***		6.197	3.058~12.588	<0.001***
**NSE (ng/ml)**							
≤25	1.000	Referent			1.000	Referent	
>25	3.522	2.019~6.146	<0.001***		1.919	1.014~3.634	0.045*
**Cyfra21-1 (ng/ml)**							
≤3.3	1.000	Referent					
>3.3	1.177	0.786~1.764	0.429				
**CEA (ug/L)**							
<5	1.000	Referent					
≥5	0.974	0.664~1.429	0.894				
**SCC (ng/ml)**							
<2.5	1.000	Referent					
≥2.5	1.096	0.507~2.369	0.816				
**Treatment mode**							
chemotherapy	1.000	Referent			1.000	Referent	
chemotherapy + radiation	0.220	0.029~1.644	0.140		0.643	0.080~5.197	0.679
surgery	0.232	0.056~0.968	0.045*		0.333	0.077~1.443	0.142
none treatment	4.219	2.702~6.588	<0.001***		4.310	2.564~7.245	<0.001***

Abbreviations: HR, hazard ratio; 95% CI, 95% confident interval; NSE, neuron-specific enolase; Cyfra21-1, cytokeratin 19 fragment; CEA, Carcinoembryonic antigen; SCC, squamous cell carcinoma associated antigen; CTC, circulating tumor cells. ^*^p<0.05, ^**^p<0.01, ^***^p<0.001.

**Table 3:** Points for variables in nomogram models.

Variables	Points		Total points	Nomogram		
	MST	OS		MST (days)	OS (probability)	
					1 year	2 year
**Age (years)**			178	200		
≤61	0	0	113	400		
62~79	31	31	71	600		
≥80	52	52	37	800		
**TNM stage**			12	1000		
I/II	0	0	8	1200		
III/IV	39	39	5	1400		
**CTC(n)**			171		0.1	
≤9	0	0	156		0.2	
10~24	23	23	144		0.3	
≥25	78	78	132		0.4	
**NSE (ng/ml)**			121		0.5	
≤25	0	0	108		0.6	
>25	29	29	93		0.7	
**Treatment mode**			74		0.8	
chemotherapy	32	32	42		0.9	
chemotherapy + radiation	15	15	102			0.1
surgery	0	0	87			0.2
none treatment	100	100	75			0.3
			63			0.4
			52			0.5
			39			0.6
			24			0.7
			4			0.8

Abbreviations: NSE, neuron-specific enolase; CTC, circulating tumor cells; MST, median survival time; OS, overall survival.
